# Physiological Factors Related to Aspiration Risk: A Systematic Review

**DOI:** 10.1007/s00455-014-9516-y

**Published:** 2014-02-23

**Authors:** Catriona M. Steele, Julie A. Y. Cichero

**Affiliations:** 1 Toronto Rehabilitation Institute – University Health Network, 550 University Avenue, #12-101, Toronto, ON M5G 2A2 Canada; 2 University of Toronto, Toronto, ON Canada; 3 Bloorview Research Institute, Toronto, ON Canada; 4 School of Pharmacy, University of Queensland, Woolloongabba, QLD 4102 Australia

**Keywords:** Swallowing, Deglutition, Deglutition disorders, Aspiration, Systematic review

## Abstract

Penetration–aspiration is considered the most serious component of oropharyngeal dysphagia. Clinicians regularly evaluate the pathophysiology of swallowing and postulate reasons or mechanisms behind penetration–aspiration. In this article we share the results of a two-stage literature review designed to elucidate the association between abnormalities in physiological measures of swallowing function and the occurrence of penetration–aspiration. In the first stage, a broad scoping review was undertaken using search terms for nine different structures involved in oropharyngeal swallowing. In the second stage, based on the results of the initial search, a more focused systematic review was undertaken which explored the association between aspiration and abnormalities in respiratory, tongue, hyoid, and laryngeal function in swallowing. A total of 37 articles underwent detailed quality review and data extraction in the systematic review. The results support measurement of tongue strength, anatomically normalized measures of hyoid movement, bolus dwell time in the pharynx while the larynx remains open, respiratory rate, and respiratory swallow phasing as parameters relevant to aspiration risk.

Aspiration, or the entry of material into the airway, is a major concern for individuals with dysphagia (swallowing impairment). A major emphasis in the evaluation of dysphagia is to identify physiological abnormalities in swallowing that contribute to or explain a patient’s risk of aspiration. Management strategies are then selected to address these contributing factors in the hope of limiting the risk of aspiration and its sequelae. To date, however, the identification of contributing factors remains subjective and inferential. This article reports a two-stage literature review process intended to elucidate pathophysiological factors that are documented to occur in association with aspiration and may provide clues regarding the underlying reasons for aspiration. In the first phase, a broad scoping review was undertaken to pinpoint physiological factors of potential relevance. In the second phase, four physiological factors were included in a more focused systematic review of the available literature on swallowing and swallowing disorders.

## Phase 1: Scoping Review

The initial scoping review was designed to identify measures and parameters that correlate with impaired swallowing safety and thus increase a person’s risk of aspiration. The framework for the scoping review approached the oropharyngeal swallowing system as a series of pumps and valves. Using this broad framework, we identified ten unique fields of research for inclusion in the search, as shown in Table 1. The esophagus was not included in this literature review.

**Table 1:** Parameters used in the scoping review search strategy

Phase of swallowing	Parameter
n/a^a^	Respiration
Oral phase	Jaw/mandible
	Lips/labial
Oral and pharyngeal phase	Soft palate/velopharyngeal
	Tongue/lingual
Pharyngeal phase	Hyoid
	Epiglottis
	Larynx
	Pharyngeal
	UES/cricopharyngeal

^a^Respiration is important before, during, and after the swallow

### Methods

To identify articles of interest, a multifield search was performed using the following search engines:

- Ovid MEDLINE^®^ 1950–2010
- Ovid MEDLINE^®^ in-process and other nonindexed citations to 2010
- AMED (Allied and Complementary Medicine) 1985–2010
- EMBASE 1980–2010
- Health and Psychosocial Instruments 1985–2010
- PsycINFO 1967–2010

Search terms included the MeSH Headings “Swallowing” OR “Deglutition” AND a title search term for each parameter. The search was limited to articles in English, dealing with human data, and published either in journals or as advanced release electronic publications between 1985 and 2010. Table 2 lists the numbers of nonduplicate articles identified for each title search term using this strategy and those that were subsequently identified as relevant for closer review, following a review of titles and abstracts for relevancy. The relevancy review was completed by two trained research assistants who were blinded to each other’s decisions. At this stage of the review, where there was disagreement regarding the relevancy of an article, it was retained for subsequent consideration. The full text of these 373 articles was then reviewed in duplicate by the same research assistants to confirm whether the article described swallowing physiology or its measurement in relation to swallowing safety or aspiration. Differences in opinion regarding relevancy were resolved by independent review by the first author, with the default rule being to include an article for further review whenever there was potential relevance. This process resulted in reduction of the set to 190 articles. These were divided into subsets according to the parameters of interest (i.e., the original search terms), with half of the articles reviewed in detail by each author. The review process involved extraction of research design, sample size, research objectives, key findings related to the association between a physiological parameter and aspiration, and a ranking of the level of evidence using the National Health and Medical Research Council of Australia ranking system (Table 3).

**Table 2:** Scoping review search results

Title search term	Search yield	Retained after relevancy check
Respiration	40	38
“Jaw or Mandib*”	81	16
Labial or Lip	34	10
“Soft Palate or Velophar*”	35	12
“Tongue or Lingua*”	257	76
Hyoid	43	26
“Epiglott*”	23	3
“Laryn*”	418	57
“Pharyn* (also captures cricophar*)”	443	135
TOTAL	1,374	373

Asterisk in the search term indicates that terms with the specified word stem will be captured, allowing for a variety of word endings

**Table 3:** Method of ranking levels of evidence, as proposed by the National Health and Medical Research Council of Australia

I	Evidence from systematic review of all relevant randomized controlled trials
II	Evidence from at least one properly designed randomized controlled trial, retrospective studies
III-1	Evidence from well-designed pseudorandomized controlled trials (e.g., alternate allocation or some other method)
III-2	Evidence from comparative studies with concurrent controls and allocation not randomized (cohort studies), case–control studies, or interrupted time series with a control group (i.e., nonconsecutive cohort study)
III-3	Evidence from comparative studies with historical control, two or more single-arm studies, or interrupted time series without a parallel control group
IV	Evidence from case series, either post-test or pretest and post-test, or superseded reference standards
V	Expert opinion, physiology, bench research or “first principles” studies

### Scoping Review Findings

The majority of articles that were found in the scoping review reported Level III, IV, or V evidence. These levels mean that the articles were predominantly studies that looked at a population of interest and performed a single evaluation to characterize swallowing function in that group (Level IV), or were comparative studies using a control group to determine whether differences were seen in persons with dysphagia compared to healthy individuals (Level III). Level V evidence came from studies of physiology, anatomy, and “first principles” analysis. In many cases the sample size was less than 10. The ability to confidently generalize to larger populations from these data is dubious. The data often had wide standard deviations, indicating variability within and between groups of healthy participants and those with dysphagia. Notwithstanding these limitations, the scoping review suggested a risk for aspiration associated with particular parameters. Advanced age (beginning at 50 years) emerged as an independent factor associated with aspiration ( [1] Level IV; [2] Level III-2). Issues related to jaw, lip, soft palate, and epiglottic, pharyngeal, and upper esophageal sphincter function were all found to have no direct, independent association with aspiration. However, dysfunction in these parameters usually occurred with abnormalities in other physiological parameters, for which the evidence regarding the association with aspiration is summarized below.

#### Respiratory Factors Associated with Aspiration

1. Abnormalities in respiratory rate and oxygen saturation ( [3] Level III-2; [4] Level IV; [5] Level III-3; [6] Level IV; [7] Level IV; and [8] Level III-2): For adults, typical resting respiratory rate is reported to be 16–20 breaths/min. Following stroke, individuals have been reported to have a faster resting respiratory rate than controls [3]. Morton et al. [4] reported that moderate to severely abnormal respiratory function was more common in aspirating patients than in nonaspirators and found that rapid and high velocity or chaotic respirations were commonly linked with aspiration. The literature on oxygen saturation suggests that drops of 4 % may be seen in people who aspirate [5, 7], while oxygenation remains stable in nonaspirators [6]. However, a time lapse of at least 5 s should be expected prior to observing potential drops in peripheral blood oxygenation measures following an aspiration event [8].
2. Any abnormality of maximal inspiratory or total lung capacity as measured via spirometry (Level IV [9] ).
3. Inconsistency in swallow-respiratory pattern between two swallows ( [10] Level III-2).
4. A swallow-respiratory phasing pattern that does not involve exhalation–apnea–exhalation ( [10] Level III-2; [11] Level III-3; [12] Level IV).
5. Short swallow apnea duration ( [12] Level IV) or variability in apnea duration between swallow trials ( [11] Level III-3).

In addition to these respiratory factors, prolonged bolus dwell time in the pharynx, prior to or following swallow, was reported in the respiratory article subset to be associated with aspiration, particularly if paired with

- Inhalation plus abnormality of respiration ( [4] Level IV; [12] Level IV), and/or
- Abnormal total lung capacity or maximal inspiratory capacity ( [4] Level IV).

Morton et al. [4] reported that for individuals who aspirated, the bolus dwelled in the pharynx significantly longer (6.2 s; 95 % CI 2.6, 9.9) than for individuals with dysphagia who did not aspirate (2.4 s; 95 % CI 1.6, 3.2). Interestingly, the authors did not differentiate between the presence of a preswallow bolus in the pharynx (presumably indicating poor oral bolus control with premature spillage and/or delayed initiation of the pharyngeal swallow) and the presence of a post-swallow bolus in the pharynx (i.e., residue).

#### Tongue Factors Associated with Aspiration

1. Reduced tongue–palate pressures ( [13] Level IV; [14] Level III-2), which are the primary driving force for bolus propulsion ( [15] Level IV; [16] Level IV).
2. Reduced ability to control the timing of tongue–palate pressure release ( [17] Level IV).

#### Hyoid Factors Associated with Aspiration

1. Reduced hyoid excursion ( [18] Level III-2).
2. Severely reduced anterior range of hyoid movement ( [19] Level IV).
3. Weak contraction of the suprahyoid muscles, given their primary role in generating superior and anterior movement of the hyoid ( [20] Level IV; [21] Level IV).

#### Laryngeal Factors Associated with Aspiration

1. Impaired laryngeal sensation:
  - presenting in the form of an absent or diminished laryngeal adductor reflex during Flexible Endoscopic Examination of Swallowing with Sensory Testing (FEEST) ( [22] Level III-2; [23] Level IV);
  - due to anesthetic nerve block of the internal branch of the superior laryngeal nerve ( [24] Level IV; [25] Level IV).
2. Impaired laryngeal sensation in combination with reduced pharyngeal contraction on a pharyngeal squeeze maneuver ( [23] Level IV; [26] Level IV; [27] Level IV).
3. Reduced movement of the larynx:
  - Toward the hyoid, such that distance between these structures, together with the supraglottic space, remains more widely open than in healthy individuals ( [28] Level IV);
  - In the anterior direction ( [19] Level IV).
4. Reduced duration of laryngeal closure in combination with delayed swallow response and prolonged pharyngeal transit time ( [29] Level III-2).

### Scoping Review Discussion

In addition to these findings, an important concept that emerged from the literature on respiratory, laryngeal, and pharyngeal function in swallowing is the idea that any single factor, when assessed in isolation, may be insufficient to accurately predict or explain aspiration as verified on videofluoroscopy [2, 30]. However, multivariate modeling that combines several risk factors may achieve good sensitivity and specificity for distinguishing aspirators from nonaspirators ( [2] Level IV; [22] Level IV; [25] Level IV; [28] Level III-2).

## Phase 2 Systematic Review Methods

**Fig. 1:**
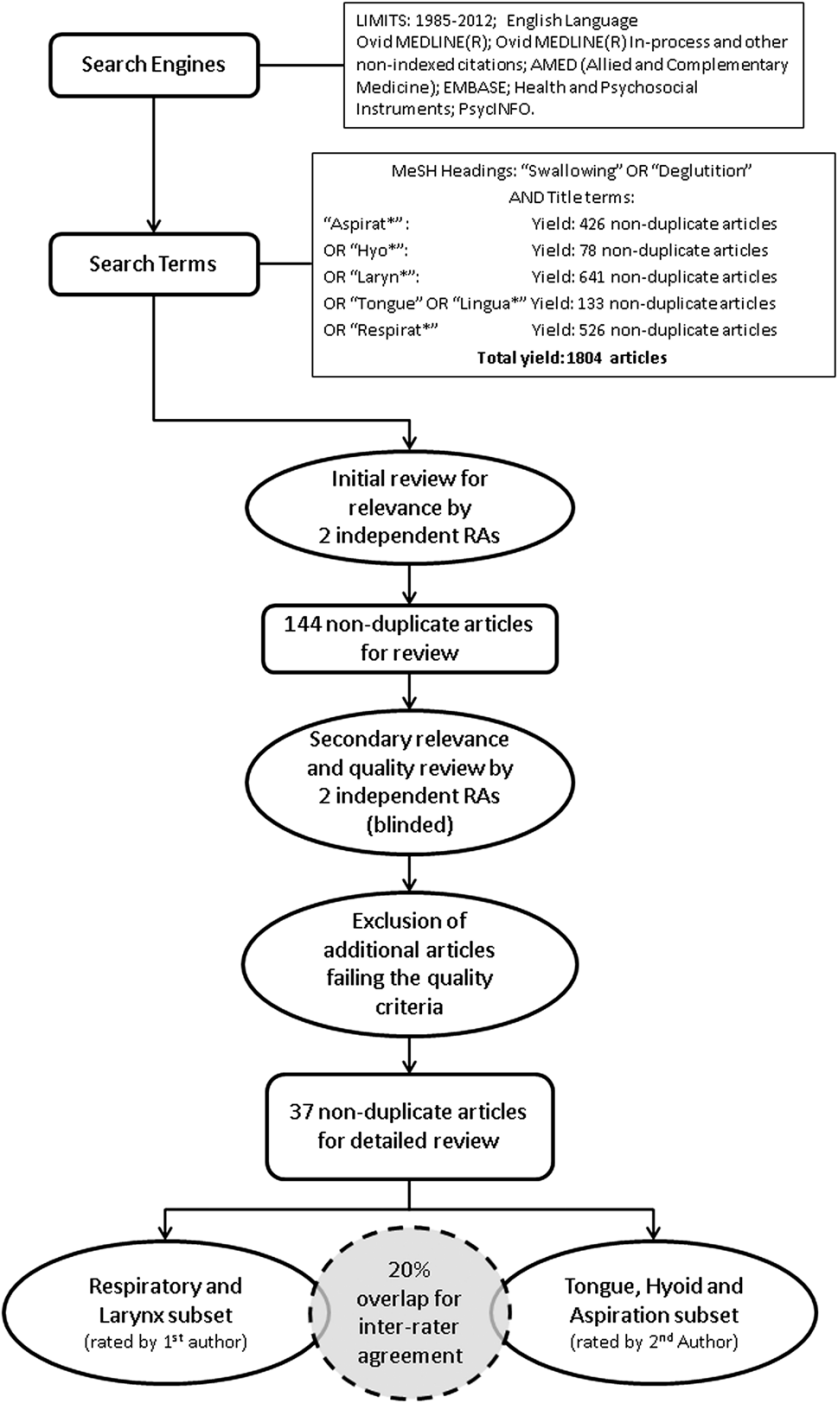
Search process for systematic review

Based on the initial scoping review results, we decided to undertake a second, more focused search, exploring respiratory, tongue, hyoid, and laryngeal factors associated with aspiration. The search strategy resembled that used for the scoping review, using the same search engines but an end date of 2012. Search terms included MeSH headings of “Swallowing” OR “Deglutition” in combination with title terms “Aspirat*” OR “Respirat*” OR “Tongue” OR “Lingua*” OR “Hyo*” OR “Laryn*” and yielded an initial set of 1,804 articles for review, which was subsequently reduced to 144 nonduplicate articles that were determined to be relevant. The search and review process is illustrated in Fig. 1.

An important addition to the review process for this second search was a review of study quality [31–33]. When an article was authored by one of the reviewers, it was assigned to the other reviewer to limit bias. The quality review process began with three questions that served as further inclusion criteria:

1. Was the participant sample representative of those who might be assessed for aspiration?
2. Was a gold-standard test (VFSS or FEES) used to confirm aspiration status?
3. Was a quantifiable parameter compared between aspirators and nonaspirators?

In the event that the answer to any of these questions was “no,” the article was excluded from further review. The process then continued with the extraction of detailed information about each study, using the following questions:

- Was the study method described in enough detail to permit replication?
- What parameter was measured and compared between aspirators and nonaspirators?
- Is it feasible to measure the parameter in a typical patient population?
- Does the study report descriptive or inferential statistics comparing groups?
- Was there a control group?
- Could the results have been influenced by knowledge of outcome (i.e., was there a lack of blinding)?
- What was the level of evidence?

The answers to these questions were put into a table that supported the comparison of article quality, methods, results, and level of evidence and informed our interpretation of the literature.

### Systematic Review Results

In general, it must be noted that the majority of the literature identified in this systematic review was found to not be of good quality. In particular, reported study methods were vague and general such that they would not permit replication. A common example of this vagueness occurred with respect to the reported details of how the instrumental swallowing assessment reference tests were performed. For example, we found several articles which indicated that either videofluoroscopy or FEES examination had been conducted to confirm aspiration status. However, no further details were provided regarding the protocol used. As such, the definition of “aspirator” might have been applied based on a single swallow of a single consistency or on multiple swallows of several consistencies. The reader was largely left to trust that these instrumental procedures must have been performed accurately in the hands of the authors or their trainees. Operational definitions of “aspirator,” or even “aspiration,” were largely lacking, and there was very limited reporting of whether patients were classified online by the clinician performing the exam or as the basis of post hoc reviewing by individuals blinded to additional information about the patient that might bias the classification. Details regarding inter- and intrarater reliability for aspiration classification or for any of the parameters explored were almost exclusively absent from the literature. A further concern that was identified regarding several of the studies selected for detailed review had to do with questionable statistical methods, in particular, the mishandling of repeated measures in analyses of variance. Notwithstanding these concerns, a number of conclusions can be made based on the reported evidence.

#### General Findings Regarding Aspiration

1. Endoscopic studies in healthy older volunteers suggest that it is not uncommon for healthy elders to aspirate ( [34] Level IV). It should be noted that the visibility or appreciation of aspiration may differ between endoscopic and videofluoroscopic studies, since the endoscopic view of the larynx is obliterated during pharyngeal constriction and endoscopy permits a more direct view of the vocal folds and tracheal rings after the swallow [35].
2. Aspiration status, rated as <3 vs. ≥3 on the penetration–aspiration Scale [36], varies across swallows within individuals, i.e., there can be variation in pattern, across a series of six thin-liquid swallows from the same person ( [29] Level III-2).
3. Some factors (e.g., the duration of laryngeal vestibule closure) appear to vary as a function of age or of having a condition such as stroke (as opposed to being a control participant or healthy volunteer) but do not differ as a function of aspiration status ( [37–39] Level III-2).
4. Age is a significant risk factor for both penetration and aspiration. Specifically, individuals over the age of 80 years are at increased risk for penetration and aspiration. This finding was found in healthy individuals and suggests that some elders may already be at increased risk prior to a neurological event or structural insult/surgery ( [34] Level IV).
5. Different bolus types involve different risk for aspiration. In healthy elders undergoing endoscopic evaluations of swallowing, 2 % and whole-fat milk elicited increased rates of penetration and aspiration over water. This finding may also be true for elders after neurological/structural insults/surgeries that affect the swallowing system ( [34] Level IV).

#### Respiratory Factors Associated with Aspiration

1. A resting respiratory rate of >25 breaths/min is associated with increased risk of aspiration in healthy individuals and those with respiratory disorders such as chronic obstructive pulmonary disease (COPD) ( [40] Level III-2).
2. Drops in oxygen saturation are not always associated with aspiration ( [40] Level III-2). However, a low baseline oxygen saturation level (<94 % SpO_2_) is associated with increased risk of aspiration ( [40] Level III-2).
3. Aspiration is more common when swallow apnea is not bracketed by an exhalation–exhalation respiratory pattern, particularly when swallowing large volumes of liquid (100 ml) ( [10] Level III-2; [11] Level III-3; [40] Level III-2).

However, one study failed to observe an abnormal pattern of postapnea inspiration in the COPD population (either aspirators or nonaspirators), for 5-, 10-, and 20-ml volumes. Therefore, the assumption that individuals with respiratory compromise necessarily display abnormal swallow–respiratory phasing is not supported ( [40] Level III-2).

#### Tongue Factors Associated with Aspiration

1. In healthy elders, both maximum isometric tongue–palate pressures and swallowing tongue pressures measured at the anterior and posterior palate are significantly reduced in those who aspirate compared to those who do not aspirate ( [34] Level IV).
2. Poor tongue driving force (inferred from videofluoroscopic measures of bolus speed) is significantly associated with aspiration in individuals with amyotrophic lateral sclerosis and in frail elders with multiple comorbidities ( [41, 42] Level IV).

#### Hyoid Factors Associated with Aspiration

1. The literature examining the relationship between hyoid movement and aspiration contains mixed reports of association, which vary depending on the unit of measurement used to capture the extent of hyoid movement:
  - Studies involving millimeter measurements of hyoid movement demonstrate no association with aspiration ( [43] Level III-3; [44] Level III-3).
  - One study ( [19] Level IV) argued that the most accurate way to measure hyoid movement is by using anatomically normalized units (i.e., as a percent of the height of a C2–C4 cervical spine scalar). This has been shown to neutralize sex and height differences that may confound measures in millimeters. When anatomically normalized units are used, markedly reduced anterior hyoid movement has been found to be associated with a greater chance of aspiration/penetration ( [19] Level IV).

#### Laryngeal Factors Associated with Aspiration

1. The opportunity for aspiration appears to increase with longer bolus dwell time in the pharynx when the laryngeal vestibule is not closed [29, 38, 39, 45] Level III-2; [4] Level IV). As mentioned in the findings of the scoping review, these studies failed to differentiate bolus presence in the pharynx prior to airway closure from residual bolus presence in the pharynx after the resumption of breathing.
2. Measures of the duration of laryngeal vestibule closure (once achieved) are not good at dissociating aspirators from nonaspirators ( [37–39] Level III-2). Laryngeal vestibule closure is defined as contact between the arytenoids and the base of the epiglottis and should not be confused with closure of the true vocal folds, which cannot be discerned from lateral view videofluoroscopy.

### Systematic Review Discussion

This review is not without limitations. It is important to recognize that reviews of this type are inherently limited by choices made in search terms and approach. In this case, the search strategy was limited to title terms and may therefore not have captured relevant articles in which the search terms were buried in the abstract or the full text. In addition, literature searches of this type are unlikely to find articles that have been published in lower-impact journals that are not yet indexed, or those articles that have been indexed using different key words. Nevertheless, based on the findings of this review, it is possible to formulate several recommendations for clinical practice with respect to measures that show promise for delineating individuals who are at risk of aspiration compared to nonaspirators. These recommendations are listed and discussed below.

1. Tongue-pressure generation (maximum isometric tongue pressures) should be measured in adults at risk for aspiration and those with reduced pressures should be referred for instrumental swallowing assessment.
  This recommendation is derived primarily from a Level IV evidence study by Butler et al. [34], in which it was reported that aspirators had significantly lower anterior and posterior maximum isometric tongue pressures than nonaspirators. Participants in this study were healthy, community-dwelling adults over 60 years old, 37 % of whom were found to display aspiration during an endoscopic examination involving nine different swallowing tasks (water and different types of milk). Maximum isometric pressures at the anterior palate had nonoverlapping 95 % confidence intervals between aspirating and nonaspirating participants, with the division between groups being between 505 and 517 mmHg (i.e., 67–69 kPa), measured using the KayPentax Digital Swallow workstation tongue bulb array. A similar divergence of scores was found for pressures measured at the posterior palate, with the boundary between groups lying between 303 and 355 mmHg (i.e., 40–47 kPa). It should be noted that in this study the maximum isometric pressure (MIP) scores reported for aspirating seniors were in the range previously reported to be normal for healthy seniors [13, 14, 46, 47]. Based on these previous studies, MIPs below 300 mmHg (i.e., 40 kPa) are typically considered to represent reduced tongue strength. The values reported by Butler et al. [34] for posterior MIPs agree with a threshold of 300 mmHg. Nevertheless, further work to replicate the findings of Butler et al. [34] is warranted before specific anterior and posterior tongue MIP thresholds related to aspiration risk can be established.
2. Measures of hyoid excursion should be calculated/expressed in anatomically normalized units to reduce artifacts attributable to size of the system.
  This recommendation arises from a single study [19] in which measures of hyoid and laryngeal excursion were expressed in values normalized to the length of the C2–C4 vertebral distance. Using this approach, the authors were able to find an association between extent of hyoid movement and aspiration status on a per-swallow basis. This finding stands in contrast to other studies that have failed to find an association between millimeter measures of hyoid movement and aspiration status. Given that hyoid movement is known to be widely variable in healthy individuals [48], methods to limit the variation that arises from data-processing decisions are desirable. Normalization of hyoid movement measurements to the cervical spine has been proposed as one method to do this [18, 19, 49].
3. It is worthwhile to measure resting respiratory rate during swallowing evaluation.
  The study by Cvejic et al. [40] found that a resting respiratory rate of 25 breaths/min was associated with aspiration in one healthy individual and individuals with COPD. For the healthy adult population, the normal resting respiratory rate is 16–20 breaths/min [50]. A higher respiratory rate provides less opportunity to obtain sufficient length of deglutition apnea. It is relatively easy to incorporate this simple measure into a clinical evaluation by placing a hand on the patient’s shoulder and clavicle and counting the breaths. Although the numbers in the study were small, the fact that this measure was consistent across both healthy individuals and individuals with respiratory compromise strengthens the argument to include the measure.
4. Desaturation events on pulse oximetry should not be interpreted as suggesting that an aspiration event has occurred.
  A number of studies, many with methodological flaws, have suggested that a drop in oxygen saturation will be time-linked with aspiration [5–8]. Physiologically, this is difficult to argue. On average, blood is pumped at a rate of 5 L/min [51]. If an aspiration event were to occur, the effect would not be instantaneous; rather, it would take up to 1 min for a drop in peripheral blood oxygenation to register. However, one must also factor in the person’s blood pressure and compliance of the arteries. These factors will affect the speed at which oxygenated or deoxygenated blood is circulated through the system and thus the likely latency before a change in oxygenation might be displayed on an oximeter. Cvejic et al. [40] found that swallowing a large liquid bolus (100 ml) was associated with transient drops in oxygen saturation in more than 80 % of the COPD population but in only 25 % of the healthy population. Swallowing a large volume of liquid typically requires a longer duration of swallow apnea. The body responds by increasing respiratory rate to compensate for this prolonged apneic period, hence the transient nature of the desaturation. Longer apnea, rather than an aspiration event, is likely the cause for a drop in oxygen saturation in these cases. Low baseline oxygen saturation was found to be significantly associated with increased risk of penetration–aspiration (<94 %). This factor is probably a more important feature in identifying potential risk.
5. Respiratory–swallow phase relationships are worth measuring during clinical swallowing assessment. When swallow apnea is not bracketed by an exhalation–exhalation respiratory pattern, instrumental assessment is warranted.
  This recommendation arises from several studies that explored respiratory–swallow phase relationships in swallowing [10, 11, 40]. These studies pointed to the possibility that a disruption in the normal exhalation–apnea–exhalation pattern may increase the risk of aspiration. We are not aware of any small, portable, validated, hand-held instrument that can be used to monitor respiratory phasing during swallowing assessment. However, where nasal cannula airflow measures or chest-wall movement measures are available, it would be of value to collect information about a patient’s respiratory patterns during swallowing assessment.
6. Measuring pharyngeal bolus dwell time when the laryngeal vestibule is open may be a good measure of aspiration risk.
  Several studies have shown that whenever a bolus is sitting in the pharynx while the larynx’s laryngeal vestibule remains open and unprotected, there is a heightened risk for aspiration [4, 29]. This situation could occur before or after the initiation of laryngeal vestibule closure during swallowing, as a result of premature bolus spillage, delayed swallow onset, or postswallow residue. The studies reviewed did not clearly differentiate between pre- and postswallow pharyngeal bolus dwell time, or between different mechanisms. Several existing measures of swallowing function approximate the phenomenon of interest. Stage transition duration, which is a measure of swallow latency, captures the interval between bolus entry into the pharynx (passing the shadow of the ramus of mandible) and the onset of hyolaryngeal excursion [39, 52, 53]. However, there is still the possibility that aspiration may occur during the time when the hyoid and larynx are moving toward their maximum position and when laryngeal vestibule closure has not yet been achieved. Consequently, with respect to preswallow aspiration risk associated with pharyngeal bolus dwell time, we propose a new parameter: bolus dwell time prior to laryngeal vestibule closure, defined as the interval between the bolus head passing the ramus of the mandible and the achievement of laryngeal vestibule closure. With respect to postswallow aspiration risk associated with residue, a recent study suggests that pixel-based measures of residue severity in the vallecular space may help to delineate thresholds above which aspiration risk increases [54]; however, since that study was performed only with thin liquids, further work is needed to fully explore the potential of this measure.

This literature review failed to find clear evidence of a relationship between the duration of upper esophageal sphincter (UES) opening and aspiration. This is somewhat surprising given that it seems logical that reduced UES opening duration might lead to postswallow residue and a heightened risk for postswallow aspiration. We have already commented on the fact that studies describing aspiration related to bolus dwell time in the pharynx while the laryngeal vestibule remains open have failed to clearly differentiate pre- from postswallow bolus presence in the pharynx. Furthermore, recent studies suggest that measures of UES opening are particularly susceptible to poor interrater agreement [53], which may threaten the ability to identify a clear pattern of association with aspiration.

## Future Directions

The results of these reviews have practical implications for increasing the chances of identifying individuals at increased risk of aspiration using instrumental and noninstrumental evaluations of swallowing. Using videofluoroscopy or endoscopy, clinicians and researchers are encouraged to use more than two swallows per bolus type to characterize an individual’s swallow, given that variation can occur in the same person over six swallows. As noted above, measures of hyoid movement using anatomically normalized units are advocated, as is measurement of the duration of bolus dwell time in the pharynx when the laryngeal vestibule is open. The inclusion of lingual manometry during instrumental testing may start to provide specific information on tongue driving force, allowing us to quantify the minimum pressures required for safe swallowing. From a clinical perspective, clinicians could begin to routinely record respiratory rate and swallow–respiratory phasing, noting whether an EE pattern is in use. Moreover, clinicians and researchers are encouraged to be mindful of what appears to be at least one set of combined factors associated with increased aspiration risk: (a) advancing age (>80 years), (b) respiratory rate greater than 25 breaths/min, (c) non-EE swallow–respiratory patterning, (d) maximum isometric tongue pressures below 300 mmHg, (e) poor tongue driving force, (f) reduced anterior hyoid movement (measured using anatomically normalized units), and (g) increased length of time the bolus dwells in the pharynx with the laryngeal vestibule open (>6 s). With improved attention to the quality of research, further understanding of the risks associated with combined factors may also emerge.

## Conclusions

In conclusion, using a two-stage systematic review with progressively narrowing focus, we have identified a number of measures relating to tongue strength, anatomically normalized hyoid movement, respiratory measures, and the length of time the bolus remains in the pharynx with the airway open as measures that are reported to demonstrate an association with increased risk of penetration–aspiration. It is important to remember that the observed associations cannot be interpreted as causative. Nevertheless, when a clinician observes aspiration in conjunction with abnormalities in these associated factors, it may guide their understanding of the reasons for or mechanisms behind aspiration and prompt the selection of interventions that are specifically intended to address these pathophysiological mechanisms.

It is important to emphasize that the studies discussed in this review typically described patterns of association in group means for the parameters of interest between individuals displaying aspiration compared to those who do not aspirate. The pathophysiology of aspiration in an individual patient may or may not correspond to trends seen at the group level. Thus, it remains important to inspect the various factors identified in this review for each patient to determine the most likely explanations for aspiration. Similarly, it is important to remember that the manner in which these parameters have been measured in the literature may fail to capture relevant information. For example, although this review failed to identify a significant association between the duration of laryngeal vestibule closure and aspiration, it may be extremely salient to know whether laryngeal vestibule closure is mistimed, either beginning late or ending early. Furthermore, given that recent evidence shows that the duration of laryngeal vestibule closure is a parameter that varies with bolus volume [53], a thorough investigation of the integrity of laryngeal vestibule closure and its association with aspiration risk should involve several different volumes of thin liquid. The literature reviewed in this study fell short with respect to thoroughly investigating these relationships.

As noted earlier, the findings of this review underscore the conclusion that it is unlikely that any one feature will reliably identify or explain aspiration risk in isolation or predominate over other risk factors with respect to its sensitivity and specificity. Rather, it is far more likely that there is a complex interaction between the identified risk factors such that a tipping point in the intricate balance that governs swallowing can shift the patient toward increased risk. One of the reviewed studies demonstrated this concept nicely, showing that increased baseline respiratory rate, lower baseline oxygen saturation, reduced hyoid elevation, and postswallow pharyngeal residue were associated with impaired swallowing and increased risk of aspiration [40]. Similarly, another study in stroke patients showed that a model in which swallow response time, pharyngeal transit time, and laryngeal vestibule closure were combined performed better at predicting aspiration status than any of these parameters alone [29]. Of the factors identified as being associated with aspiration in this review, reduced tongue strength is the most easily measured given currently available low-technology instruments. Tongue strength also identifies itself as a logical target for therapy, with the potential to reduce aspiration risk.

Finally, it was disappointing to discover a large number of papers of poor methodological quality and therefore did not qualify for inclusion in this review. Even those articles that did meet both our inclusion and quality criteria had limitations and flaws in methodology and design, which means that we are unable to formulate strong conclusions regarding the different pathophysiological presentations that are linked to a risk of aspiration. In order to advance knowledge in the dysphagia field, scientists must be vigilant in the quality of their research design and reporting.
